# Supplementary material for: Formal Definitions of Unbounded Evolution and Innovation Reveal Universal Mechanisms for Open-Ended Evolution in Dynamical Systems
Source: Sci Rep. 2017 Apr 20;7:997. doi: 10.1038/s41598-017-00810-8 (PMC5430523; doi:10.1038/s41598-017-00810-8)
Supplement: Supplementary file 1 — Supplementary info [file 41598_2017_810_MOESM1_ESM.pdf]

Supporting Information: Formal Definitions of Unbounded Evolution and Innovation Reveal Universal Mechanisms for Open-Ended Evolution in Dynamical Systems

Alyssa Adams[1,2,3], Hector Zenil[3,4,5], Paul C.W. Davies[1], and Sara Imari Walker[1,6,7,8,*]

[1]Beyond Center for Fundamental Concepts in Science, Arizona State University, Tempe AZ USA
[2]Department of Physics, Arizona State University, Tempe AZ USA
[3]Algorithmic Nature Group, LABORES, Paris, France
[4]Department of Computer Science, University of Oxford UK
[5]Information Dynamics Lab, SciLifeLab, Department of Medicine Solna, Karolinska Institute, Stockholm Sweden
[6]School of Earth and Space Exploration, Arizona State University, Tempe AZ USA
[7]ASU-SFI Center for Biosocial Complex Systems, Arizona State University, Tempe AZ USA
[8]Blue Marble Space Institute of Science, Seattle WA USA
[*]To whom correspondence should be addressed: sara.i.walker@asu.edu

ABSTRACT

Supporting information and techincal details for the manuscript *Formal Definitions of Unbounded Evolution and Innovation Reveal Universal Mechanisms for Open-Ended Evolution in Dynamical Systems*.

1 Description of Implementations of Time-Dependent Cellular Automata Variants

We consider three new variants of cellular automata (CA) to identify mechanism(s) that can produce conditions necessary for open-ended evolution (OEE) in bounded regions, subject to the formal criteria for OEE laid out in Definitions 1 and 2 in the main text. We consider definitions of unbounded evolution (UE) and innovation (INN) that are applicable to *any* instance of a dynamical system u that can be decomposed into two interacting subsystems o and e. Each CA variant implements *time-dependent* rules for o, with different functional forms f for this time-dependence. Here we describe in detail the implementation of each variant considered.

1.1 Case I: Deterministic State-Dependent Rules in Subsystem o

The first variant, Case I, implements *state-dependent* update rules. Case I CA are composed of two spatially separate, fixed-width, 1-dimensional CA: an "organism" o and an environment e. Both o and e are implemented with periodic boundary conditions, and utilize the alphabet $\{0,1\}$. The environment e is an execution of an ECA, and is evolved according to a fixed rule drawn from the set of 256 possible ECA rules, with periodic boundary conditions.

The subsystem o updates its rule according to a function f such that $r_o(t+1) = f(s_o(t), r_o(t), s_e(t))$, where s_o and r_o are the state and rule of the organism and s_e is the state of the environment. It is evolved with periodic boundary conditions. The expressed ECA rule of o at time t, $r_o(t)$, is represented by the eight-bit binary representation of its rule table[1], *e.g.* an o implementing Rule 30 at time t will have $r_o(t) = [0,0,0,1,1,1,1,0]$ (see main text Fig. 3). We refer to individual bits within the rule by the index i such that $r_o(t)[1] = 0$, $r_o(t)[2] = 0$, $r_o(t)[3] = 0$, $r_o(t)[4] = 1$ *etc.* for an o implementing Rule 30 at time t. The binary representation of ECA rules are structured such that each successive bit i iterated in this manner represents the output of application of the rule to the ordered set of triplet states $S^3 = [111,110,101,100,011,010,001,000]$.

The function f for our example implementation of state-dependent CA is constructed such that at each time-step t it compares the normalized frequency of each triplet i in S^3 in the state of o and e, $s_o(t)$ and $s_e(t)$, respectively, and flips the corresponding bit i in $r_o(t)$ if i is expressed in s_o and the normalized frequency of the triplet in $s_o(t)$ meets or exceeds the normalized frequency in $s_e(t)$ (where the frequency is normalized relative to the number of possible triplets in the state). That is, at each time-step t, a bit i in $r_o(t)$ will flip $0 \leftrightarrow 1$ if $n_i(s_o(t)) \geq n_i(s_e(t))$, where n_i counts the relative frequency of triplet i. Formally,

$$r_o(t+1)[i] = \begin{cases} \overline{r_o(t)[i]} & \text{if} \quad n_i(s_o(t)) \geq n_i(s_e(t)) \\ r_o(t)[i] & \text{if} \quad n_i(s_o(t)) < n_i(s_e(t)) \end{cases} \tag{1}$$

where the overbar represents logical negation.

An example implementation of this update function is shown in Fig. 4 in the main text, where an "organism" o with $w_o = 4$ is coupled to an environment e with $w_e = 6$, and $r_o(t) = [0,0,0,1,1,\underline{1,1,0}]$. In the example, only for $i = 3$, corresponding to the triplet $\{1,0,1\}$, is $n_3(s_o(t)) \geq n_3(s_e(t))$. Therefore, $r_o(t+1)[3] = \overline{r_o(t)[3]} = \bar{0} = 1$, as shown schematically in Fig. 5 in the main text. In this example, the interaction of o and e under f changes r_o from Rule 30 at time-step t to Rule 62 at $t+1$.

1.2 Case II: Deterministic Time-Dependent Rules in Subsystem o

The second variant, Case II, is similarly composed of two spatially separate, fixed-width, 1-dimensional CA: an "organism" o and an environment e. As with Case I, both o and e are implemented with periodic boundary conditions, and utilize the alphabet $\{0,1\}$. The environment e is an execution of an ECA, and is evolved according to a fixed rule drawn from the set of 256 possible ECA rules, just as in Case I.

The key difference between Case I and Case II CA is that for Case II, the subsystem o updates its rule according to a function f such that $r_o(t+1) = f(s_e(t))$. That is, for Case II the update rule of o depends *only* on the state of the external environment s_e and is independent of the current state or rule of o (that is, o is *not* self-referential). Formally,

$$r_o(t+1)[i] = s_e(t)[i] \tag{2}$$

Here $r_o(t)$ is determined uniquely by $s_e(t)$, such that the binary representation of each possible state of the environment uniquely maps to one ECA rule according to Wolfram's binary classification scheme[1]. For this implementation the environment must be of width $w_e = 8$ to mediate a bijective map between $\{s_e\}$ and $\{r_o\}$. Case II CA emulate systems where the rules for dynamical evolution are modulated exclusively by the time evolution of an external system.

1.3 Case III: Stochastic Time-Dependent Rules in Subsystem o

The final variant, Case III, is composed of a single, fixed-width, 1-dimensional CA: the "organism" o. Like Case II, the rule evolution of Case III is driven *externally* and does not depend on s_o. However, here the external environment e is stochastic noise and not an ECA. In Case III CA, the subsystem o updates its rule according to a function f such that $r_o(t+1) = f(r_o(t), \xi)$, where ξ introduces random fluctuations in the implemented rule of o by stochastically flipping bits in r_o. Formally,

$$r_o(t+1)[i] = \begin{cases} \overline{r_o(t)[i]} & \text{if} \quad \xi < \mu \\ r_o(t)[i] & \text{if} \quad \xi \geq \mu \end{cases} \tag{3}$$

where μ is a fixed threshold for flipping between $[0,1)$, and ξ is a random number drawn from the interval $[0,1)$. This implements a diffusive-random walk through ECA rule space. Since the rule of o at time $t+1$, $r_o(t+1)$, depends on the rule at time t, $r_o(t)$, the dynamics of Case III CA are path-dependent in a similar manner to Case I (both rely on flipping bits in $r_o(t)$, where Case I do so deterministically as a function of s_o and s_e, and Case III do so stochastically).

2 Experimental Methods

The number of possible executions grows exponentially large with w_o, limiting the computational tractability of statistically rigorous sampling of the dynamics of each CA variant and of generating the set of counterfactual isolated ECA trajectories. We therefore explored small CA with $w_o = 3, 4, \ldots 7$ and sampled a representative subset of all possible trajectories for each w_o (see Section 9 for examples of larger CA). We then generated statistics on the number of sampled trajectories satisfying Definitions 1 and 2 for unbounded evolution and innovation, respectively.

2.1 Case I Experiments

For Case I, w_e must be specified in addition to w_o. We consider systems with $w_e = \frac{1}{2}w_o$, w_o, $\frac{3}{2}w_o$, $2w_o$ and $\frac{5}{2}w_o$. For comparison to Case II and Case III CA, $w_e = w_o$ statistics are used. For each w_o and w_e, the initial state of o, $s_o(0)$, the initial state of e, $s_e(0)$, the initial rule of o, $r_o(0)$ and the rule of e, r_e, are drawn at random. For $r_0(0)$ and r_e, we only consider the 88 non-equivalent ECA rules, which dramatically reduces the number of possible cases, but still covers the full spectrum of complexity in initial configurations. We then permit r_o to evolve into any of the 256 possible ECA rules. We also ensure that no two cases sampled are initialized with the same tuple $\{s_o(0), s_e(0), r_o(0), r_e\}$.

Table 1. The size of the randomly sampled subspace for Case I CA for each w_o and w_e explored.

CA Variant	w_o	#u	% Explored	CA Variant	w_o	#u	% Explored
Case I: $w_e = \frac{1}{2}w_o$	3	2.1×10^6	1.25	Case I: $w_e = 2w_o$	3	3.36×10^7	6.92×10^{-2}
	4	4.19×10^6	1.25		4	2.68×10^8	1.73×10^{-2}
	5	1.68×10^7	0.62		5	2.15×10^9	4.69×10^{-3}
	6	3.36×10^7	0.62		6	1.72×10^{10}	3.16×10^{-4}
	7	1.34×10^8	0.31		7	1.37×10^{11}	7.75×10^{-5}
Case I: $w_e = w_o$	3	4.19×10^6	0.63	Case I: $w_e = \frac{5}{2}w_o$	3	6.71×10^7	3.91×10^{-2}
	4	1.68×10^7	0.31		4	1.074×10^9	4.88×10^{-2}
	5	6.71×10^7	0.16		5	8.59×10^8	1.22×10^{-3}
	6	2.68×10^8	7.81×10^{-2}		6	1.37×10^{11}	1.53×10^{-4}
	7	1.07×10^9	3.91×10^{-2}		7	1.1×10^{12}	3.81×10^{-5}
Case I: $w_e = \frac{3}{2}w_o$	3	8.34×10^6	0.28				
	4	6.71×10^7	6.92×10^{-2}				
	5	2.68×10^8	3.75×10^{-2}				
	6	2.15×10^9	2.52×10^{-3}				
	7	8.59×10^9	1.24×10^{-3}				

The space of all possible Case I CA executions is too large to explore the full space computationally. Since each e and o are each initiated with a state and a rule, the number of possible executions is:

$$N_U = N_R^2 \times N_{S_e} \times N_{S_o} = 88^2 \times 2^{8w_e} \times 2^{8w_o} \quad (4)$$

where N_R is the number of sampled initial rules for o and e, N_{S_e} is the number of sampled initial states for e, and N_{S_o} is the number of sampled initial states for o. For $w_o = w_e = 3$ and $w_o = w_e = 4$, exploring the full space of all possible initial conditions is computationally tractable, and verifies that the statistics reported herein for executions of o that display UE and INN are characteristic of the full computational space for the smaller sample sizes implemented in this study. The number of randomly sampled cases for Case I CA included herein is given in Table 1.

2.2 Case II Experiments

For Case II, $w_e = 8$ for all simulations, since this permits a bijective map from $\{s_e\}$ to the rule space of ECA and thus the set of rules $\{r_o\}$. As with Case I CA, executions are initialized with a randomized tuple $\{s_o(0), s_e(0), r_o(0), r_e\}$, ensuring that no two experiments are initialized with the same tuple. We restrict attention only to $w_e = 8$ for Case II experiments in this study to directly compare to our Case I and Case III CA. The number of randomly sampled cases for Case II CA is given in Table 2.

Table 2. The size of the randomly sampled subspace for Case II CA for each w_o explored.

CA Variant	w_o	#u	% Explored
Case II: $w_e = 8$	3	1.34×10^8	2.31×10^{-2}
	4	2.68×10^8	2.1×10^{-2}
	5	5.37×10^8	2.1×10^{-2}
	6	1.07×10^9	2.1×10^{-2}
	7	2.15×10^9	1.98×10^{-2}

2.3 Case III Experiments

For Case III, a threshold μ for stochastic flipping of the bits in the rule table of o must be set. Results for Case III are given for $\mu = 0.5$ in the main paper, such that each outcome bit in the rule table at every time step $r_o(t)$ has a 50% probability of flipping. Results for other values are reported in Section 5. Since we evolve only the subsystem o for Case III CA, executions

are initialized with a random tuple $\{s_o(0), r_o(0)\}$. We do not restrict sampled executions to unique tuples, since a different random seed is set for each execution. The number of randomly sampled cases for Case III CA is given in Table 3.

Table 3. The size of the randomly sampled subspace for Case III CA for each w_o explored.

CA Variant	w_o	#u	% Explored
Case III: Random	3	5.24×10^5	10
	4	1.05×10^6	5
	5	2.1×10^6	5
	6	4.19×10^6	5
	7	8.39×10^6	5

3 Calculating Recurrence Time, Compressibility and Lyapunov Exponent

Recurrence times for the state- and rule-trajectory of o were calculated to identify cases exhibiting UE and thus OEE. The complexity of the state trajectory $\{s_o(0), s_o(1), \ldots s_o(t_r)\}$ was measured by means of its *compressibility* (C), and calculation of the *Lyapunov exponent* (k).

3.1 Recurrence Time

For Cases I and II,we measured the recurrence times t'_r and t_r for o, for both the rule evolution $\{r_o(t_1), r_o(t_2), r_o(t_3) \ldots r_o(t'_r)\}$ and the state evolution $\{s_o(t_1), s_o(t_2), s_o(t_3) \ldots s_o(t_r)\}$, respectively. Recurrence times were calculated by determining the time t_r or t'_r when the *sequence* of states or rules of o, respectively, repeated. In general, t_r and t'_r for o are not the same as for the full system u (or as each other, such that often $t_r \neq t'_r$, see Fig. 1 in the main text). We therefore first determined when u repeated the tuple $\{s_o, s_e, r_o\}$ such that $\{s_o(t'), s_e(t'), r_o(t')\} = \{s_o(t), s_e(t), r_o(t)\}$, where $t < t'$. We then determined the t_r such that $\{s_o(t_r), s_o(t_r+1), \ldots s_o(t')\} = \{s_o(t_i), s_o(t_i+1), \ldots s_o(t_r)\}$ for $t_i < t_r$ (and likewise for t'_r with the replacement r_o for s_o). The time step t_i is identified as initiation of the attractor dynamics for o. In many cases, we find attractors that are unbounded and innovative by Definitions 1 and 2, in addition to full trajectories up to recurrence. An example illustrating the expected Poincaré time for o, t_P, its recurrence time for the state trajectory t_r and the attractor size for the full system u, t_a (up to the recurrence time t' for the full system) is shown in Fig. 1.

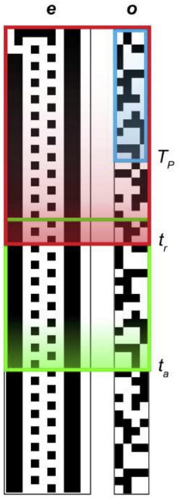

Figure 1. Relevant timescales for describing the dynamics of o embedded in u. Shown are the Poincaré recurrence time t_P (blue) for an isolated ECA of the same width w_o as o, the state-trajectory recurrence time t_r of o (red), and attractor size of the full system u, t_a (green).

Since Case III CA are stochastically evolved, their dynamics do not repeat with a unique recurrence time t_r for o. However, all executions sampled eventually terminated in an oscillation between the two homogeneous states (all-'0's or all-'1's). These states are attractors for every fixed rule ECA evolved under periodic boundary conditions, so once a Case III CA evolves to

either homogeneous state, no heterogeneity will ever be produced (the dynamics behave somewhat like dissipation of the heterogeneity in the initial state). We therefore consider it more meaningful to calculate the number of time steps before convergence to this oscillatory attractor in place of the recurrence time t_r, which we denote by t_r for consistency of notation with other cases explored. We therefore capture the timescale of relevance for all interesting (and potentially complex) dynamics, which occur in the transient before converging to this attractor.

3.2 Compressibility

The Kolmogorov-Chaitin complexity of string s is defined as the size of the shortest computer program p running on a universal Turing machine U that produces the string s (here s is the sequence of states of o):

$$K_U(s) = min\{|p|, U(p) = s\} \; . \tag{5}$$

Although it cannot be computed exactly, it is lower semi-computable and can be approximated by using a general lossless compression algorithm L[2]. This upper-bound approximation of the Kolmogorov-Chaitin complexity is normalized according to a normalized compression measure C:

$$C(s) = \frac{L(s)}{max(C_i(s), length(s))} \; . \tag{6}$$

Throughout this paper C_i is output of the Compress algorithm based on the LZW algorithm[2]. It can be replaced by the output of any other compression algorithm. The measure is therefore a family of possible indexes approximating K. We use C as measure over the state-trajectory of the organism o for each execution u, as an approximation of the characteristic complexity of o in the limit of large times $t \to \infty$.

Large values of C indicate low Kolmogorov-Chaitin complexity, meaning the output can be produced by a simple (short) program p. The normalization constant $max(C(s), length(s))$ was calculated by measuring the number of bits resulting from a generalized compression algorithm for the Poincaré recurrence time of the entire system u, not an isolated organism. This allows normalizing the observed C to its maximum possible value for an organism coupled to an environment. This closely approximates an upper limit in C for the longest possible non-repeating trajectory for any given o.

In order to ensure the normalization constant for an organism of width w_o is a close approximation to the maximal value, $C_i(s)$ was calculated for 10^7 randomly generated ECA of width w_o, evolved with a fixed rule for 2^{2w} time steps, where w is the width of u, such that $w = w_o + w_e$. The maximum of this set was used as the normalization constant $max(C_i(s), length(s))$. Thus, all C values are normalized relative to the maximal complexity of a CA evolved according to a fixed dynamical rule.

3.3 Lyapunov Exponent

The Lyapunov exponent k captures the speed at which a perturbation moves through a system[3], thereby quantifying sensitivity to initial conditions. In CA, k can be, in general, measured by perturbing a single bit in the initial condition, and counting how many bits differ compared to the unperturbed time evolution in each time step:

$$y(t) = H_{io}[s_i(t), s_o(t)] \tag{7}$$

where H_{io} is the Hamming distance between the state of the perturbed system i and the original organism o, which is evaluated at each time step t. The resulting time series of $y(t)$ values can be approximated as an exponential function, $y(t) = e^{kt}$, where k is estimated numerically. High values of k indicate sensitivity to perturbations, which is typically associated with complex dynamical systems, such as those that occur in deterministic chaos.

4 Statistics of Sampled Trajectories Displaying Innovation (INN)

Tables 4 and 5 show the resulting statistics for sampled o that were found to be innovative (INN) according to Definition 2. Table 4 includes all three CA variants as well as ECA counterfactual trajectories used as a control. Table 5 shows results for state-dependent Case I CA as a function of varying environment size w_e. Innovative o were identified as having a state-trajectory that cannot be reproduced by any closed, fixed rule ECA of equivalent width $w = w_o$.

5 Recurrence time frequency distributions

The frequency distribution of t_r observed for sampled state trajectories of o in Case I, Case II and Case III CA are shown in Figures 2,3 and 4, respectively. Comparing the three cases reveals that for equivalently sized ensembles of sampled trajectories for Case I, Case II, and Case III CA, the Case I CA generate OEE cases with higher statistical certainty than either the Case II or

Table 4. Percentage of sampled cases displaying INN for each CA variant.

w_o	ECA	Case I ($w_o = w_e$)	Case II	Case III
3	0	54.62	99.98	99.82
4	0	74.66	99.97	99.87
5	0	92.56	99.97	99.92
6	0	88.14	99.97	99.94
7	0	97.14	99.97	99.97

Table 5. Percentage of sampled cases displaying INN for Case I, with varying environment size w_e.

w_o	$w_e = \frac{1}{2}w_o$	$w_e = w_o$	$w_e = \frac{3}{2}w_o$	$w_e = 2w_o$	$w_e = \frac{5}{2}w_o$
3	30.72	54.62	70.10	86.04	93.29
4	33.32	74.66	86.57	95.52	97.47
5	32.42	92.56	96.22	98.32	98.72
6	35.64	88.14	97.03	98.91	99.29
7	52.92	97.14	97.43	99.51	99.63

Case III CA for most parameters explored. This is especially true for cases where $w_e > w_o$ in Case I simulations. From Figure 2 it is evident that larger environments yield more UE cases with $t_r > t_P$ and in general result in longer observed recurrence times.

Case II CA yield fewer OEE cases as w_o increases, as evident in Figure 3. As discussed in the main text, Case II is not scalable as it would require changing the structure of the rules of the organism o.

Case III CA generate fewer OEE cases than Case I as the width of o increases, with no cases observed in our statistical sample for $w_o > 7$ for $\mu = 0.5$ (Figure 2 bottom panel, leftmost column). The frequency distribution of recurrence times for Case III CA with $\mu = 0.01$, $\mu = 0.1$ and $\mu = 0.5$ are shown in Figure 2. For smaller values of μ OEE cases are observed for larger w_o. In the context of biological evolution, the mechanism for increasing the number of OEE cases under Case III would therefore be for systems to evolve toward *slower* mutation rates over time. However, because the distributions are exponentially distributed, the number of OEE cases is always exponentially suppressed, representing only a small tail of the distribution. A fixed width w_o execution could always be found in a *large enough* statistical sample that such that the observed t_r would be greater than the maximum recurrence time observed in a Case I CA with an equivalent organism width w_o. However, in general due to the exponential suppression of cases with larger t_r for Case III variants (Figure 4), the ensemble size of sampled cases will necessarily be much larger for Case III CA than for Case I CA. That is, for a sufficiently large ensemble size one could chose a value of μ and generate a trajectory in a width w_o organism with a given recurrence time t_r, but would always be able to find an example trajectory of the same t_r for a *smaller* sized ensemble of Case I CA for some environment width w_e. Due to the exponential suppression, OEE cases are much rarer for Case III than Case I CA. Additionally, once Case III reach the terminal attractor their dynamics are not complexity, whereas Case I CA will repeat an attractor state that is in general complex and is often times open-ended (such that the attractor itself satisfies Definitions 1 and 2). We therefore regard Case III to not be scalable.

6 ECA Rule Complexity of Case I CA

To determine if the complexity observed in Case I CA is *intrinsic* to the state-dependent mechanism, or is an artifact of a selection-effect favoring complex rules, we determined the frequency of rules implemented in Case I CA utilizing the Wolfram classification scheme for Elementary Cellular Automata[1]. There are four Wolfram Classes: Class I and II are regarded as the least complex, often generating simple repeating patterns. Class III rules are more complex displaying random patterns, and Class IV are regarded as the most complex, displaying rich dynamical structure (for example, ECA Rule 110, which is known to be Turing Universal[4] is a Class IV ECA). We analyze the complexity of ECA rules implemented in the rule trajectories of Case I CA by considering the frequency of implementation of rules from each class to determine if the complexity of the observed dynamics is an artifact of the ECA rules or *intrinsic* to f.

The resulting rank ordered frequency distribution of rules is shown in Figure 5 for all sampled Case I CA of a given organism width w_o, and separately for the OEE cases in Figure 6. Since this data includes statistics for the entire sample of o of a given width w_o included in our study, we call these distributions "metagenomes" to indicate that they represent bulk statistics over many instances of "organisms" o. The resulting distributions indicate that Case I CA primarily implement Class I and II rules, indicative that the complexity observed is *intrinsic* to the state-dependent mechanism and not an artifact of selective use of 'complex' Class III and IV ECA rules. This is true for statistics sampled over all Case I CA (Figure 5), as well as isolating only OEE cases (Figure 6).

7 Distributions of Attractor Sizes

Figures 7 - 8 show box-whisker plots of the attractor sizes for the subsystem o for each ECA and for Case I and Case II CA (Case III CA terminate in a random, oscillatory attractor and the statistics are therefore not included here, see Section 3.1 for discussion). In each figure, the black horizontal line indicates where $t_r/t_P = 1$, where t_a is the attractor size and t_P is the expected Poincaré time of an equivalent isolated system (an ECA). Sampled attractors exhibiting unbounded evolution (UE) have $t_a/t_P > 1$ and therefore fall above the black solid line - these are examples of OEE attractors.

8 Compressibility and Lyapunov Exponent Values for Case I and II CA

8.1 Compressibility and Lyapunov exponent for OEE trajectories sampled from Case I CA

Calculated values for compressibility (C) and Lyapunov exponent (k), as defined in Section 3, are shown in Figure 10 for the state trajectory of o for all sampled OEE executions for Case I CA. Comparison of the left panel of Figure 10 with the left panel of Figure 2 in the main text reveals that the observed C for all OEE cases tends to be lower than that calculated over all sampled o for Case I: that is, the OEE cases exhibit lower C, consistent with intuition that systems with longer recurrence times should be 'more complex'. As w_o increases, more OEE cases tend to have lower C values, such that larger "organisms" are more complex.

Likewise, comparing the right panel of Figure 10 with the right panel of Figure 2 in the main text indicates that OEE cases also tend to have much higher k values than that calculated over all sampled o for Case I, indicative of greater sensitivity to perturbations in OEE systems. Large values of w_o lead to larger k, on average, such that larger OEE "organisms" are more sensitive to perturbations and therefore display richer, more complex dynamics.

8.2 Comparison of compressibility and Lyapunov exponent for Case I and Case II CA

Calculated values for compressibility (C) and Lyapunov exponent (k), as defined in Section 3, are compared for Case I and Case II CA in Figures 11 and 12. Case III is not considered as the long-term dynamics display low complexity for the oscillatory attractor of the homogenous all-'0' and all-'1' states. For both Case I and Case II CA variants, increasing w_o yields more OEE cases with lower C values, such that larger "organisms" are more complex (Figure 11).

Case II CA yield lower C values than Case I for the data shown as a result of the difference in the normalization implemented in Eq. 6, which for Case II CA is lower since the the width of u is $w = w_o + 8$ for all w_o explored, whereas for Case I CA the width of u is $w = 2 * w_o$ (for $w_e = w_o$ as shown). Additionally, as noted Case I is scalable as w_e can be increased to generate higher complexity (lower C) cases. The Lyapunov exponent for Case II is in general higher than for Case I, indicating greater sensitivity to perturbations in the initial condition for Case II CA than Case I. For both CA variants, k increases with increasing organism size k, such that larger organisms are more complex.

9 Larger Systems

Figure 13 shows example executions of Case I state-dependent CA for large organisms of width $w_o = 101$, which visually demonstrate that the novelty of the dynamics reported herein scale to large system sizes.

References

1. Wolfram, S. *A New Kind of Science*, vol. 5 (Wolfram Media Champaign, 2002).
2. Zenil, H. & Villarreal-Zapata, E. Asymptotic behaviour and ratios of complexity in cellular automata rule spaces. *International Journal of Bifurcation and Chaos* **13** (2013).
3. Tisseur, P. Cellular automata and lyapunov exponents. *Nonlinearity* **13**, 1547 (2000).
4. Cook, M. Universality in elementary cellular automata. *Complex systems* **15**, 1–40 (2004).

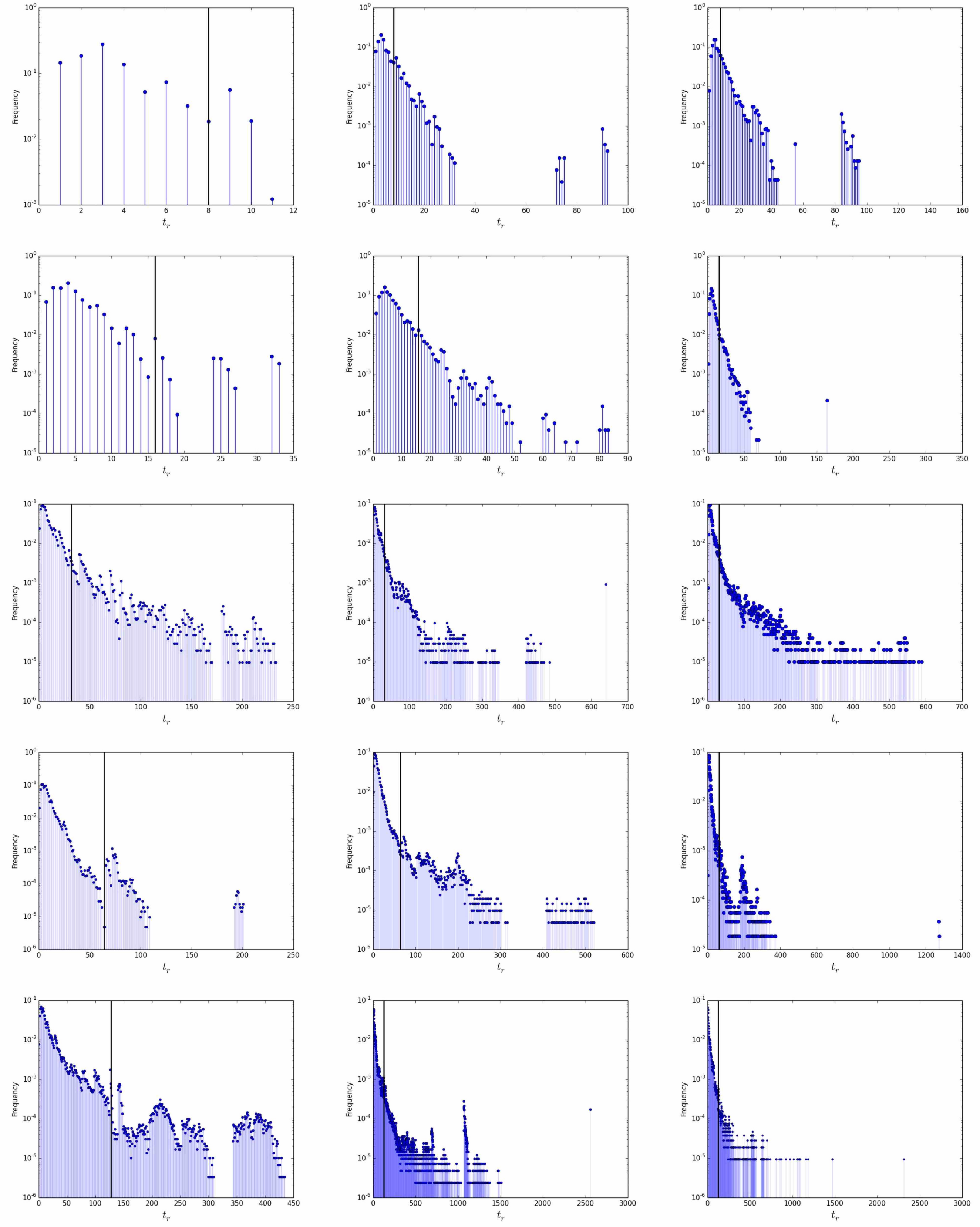

Figure 2. Frequency distributions of recurrence times t_r for Case I CA with $w_e = w_o$ (leftmost column), $w_e = \frac{3}{2}w_o$ (left middle), $w_e = 2w_o$ (right middle) and $w_e = \frac{5}{2}w_o$ (rightmost column). For rows from top to bottom, $w_o = 3, 4, 5, 6$ and 7 respectively. The Poincaré recurrence time t_P of an isolated ECA of width w_o is highlighted by the black vertical line in each panel.

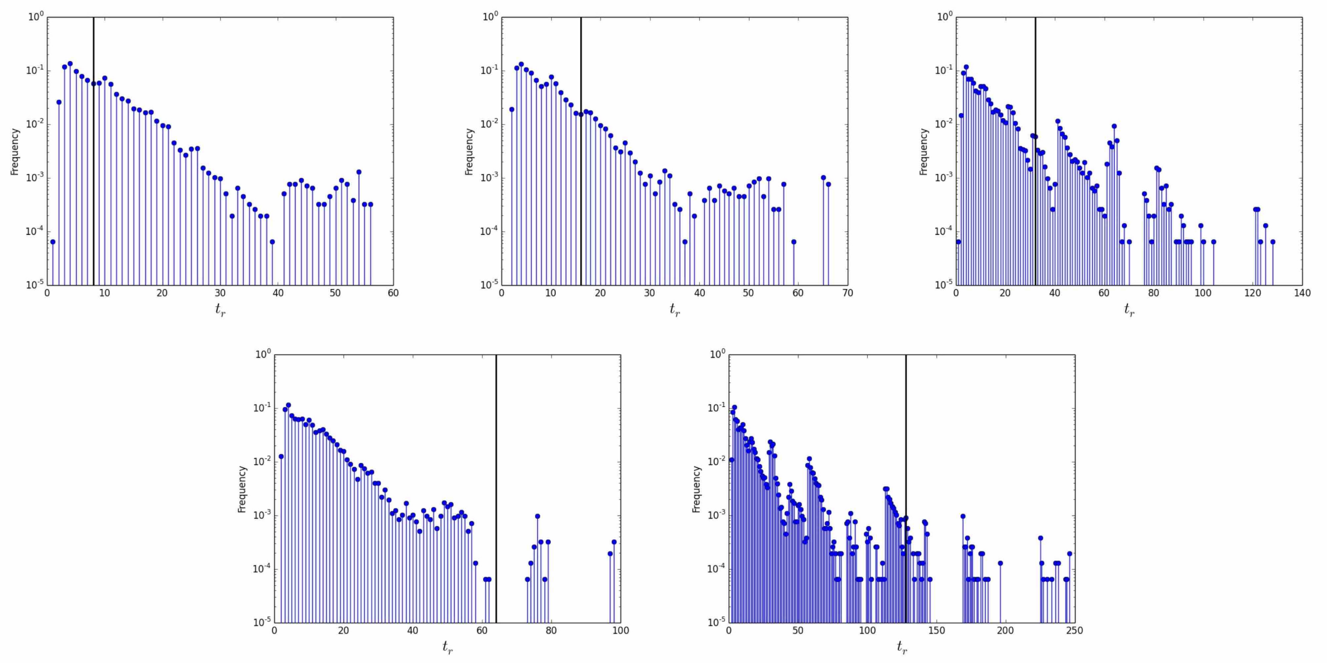

Figure 3. Frequency distributions of recurrence times t_r for Case II CA. From top to bottom, $w_o = 3, 4, 5, 6$ and 7 respectively. The Poincaré recurrence time t_P of an isolated ECA of width w_o is highlighted by the black vertical line in each panel.

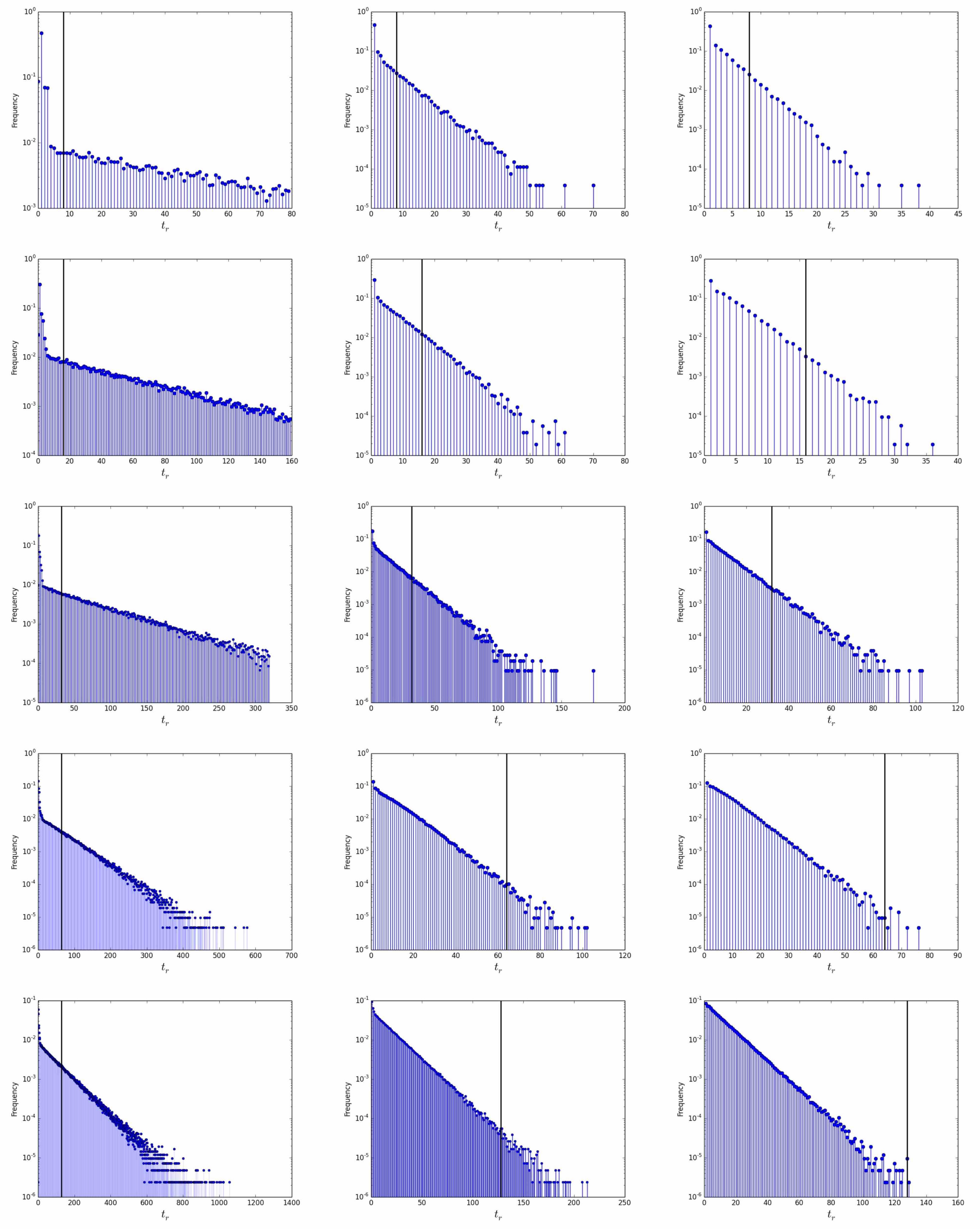

Figure 4. Frequency distributions of t_r for Case III CA with $\mu = 0.01$ (left), $\mu = 0.1$ (middle) and $\mu = 0.5$ (right). From top to bottom, $w_o = 3,4,5,6$ and 7 respectively. The Poincaré recurrence time t_P of an isolated ECA of width w_o is highlighted by the black vertical line in each panel.

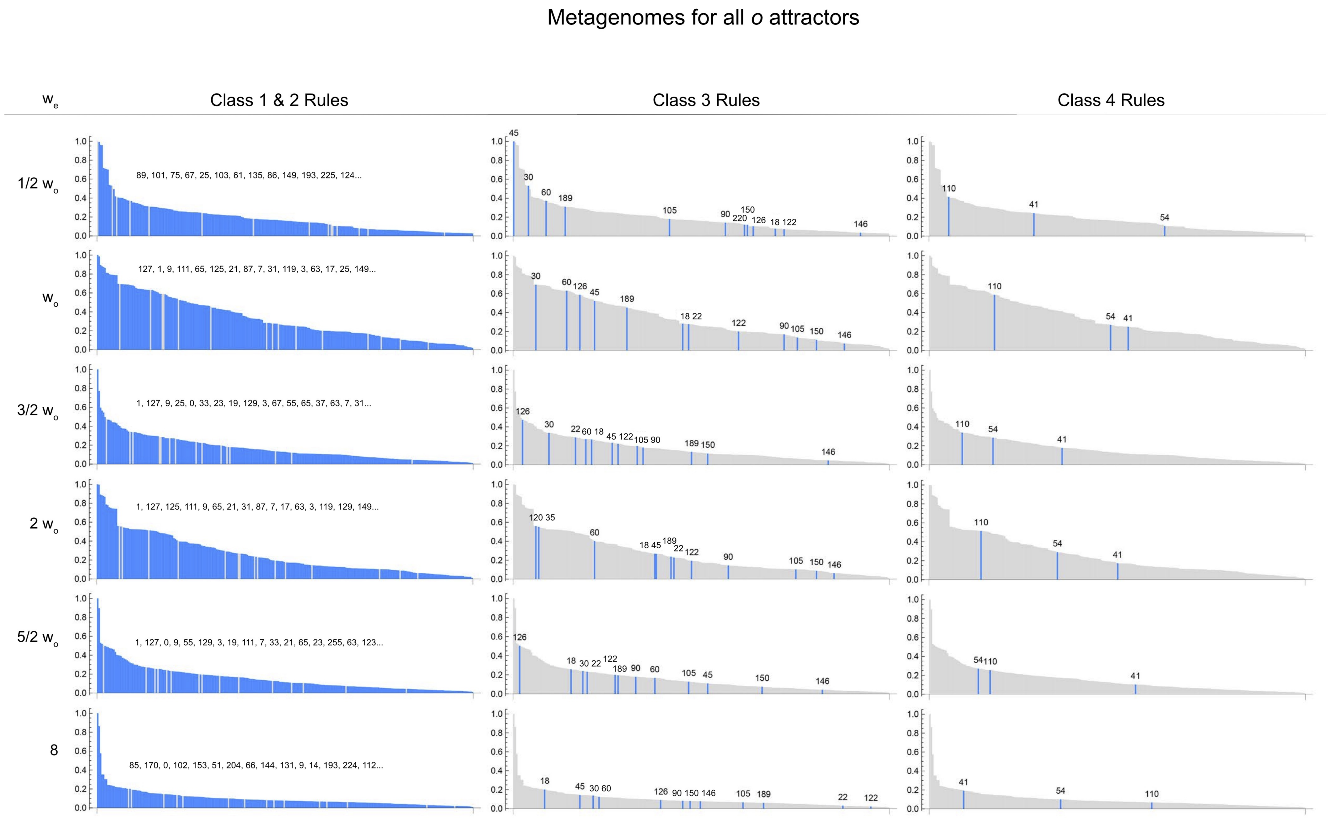

Figure 5. Rank ordered frequency distributions of rules ("metagenomes") implemented by o in its attractor. From top to bottom $w_e = \frac{1}{2}w_o$, w_o, $\frac{3}{2}w_o$, $2w_o$ and $\frac{5}{2}w_o$, respectively. Highlighted in blue are the frequencies of Class I and II rules (left), Class III rules (middle) and Class IV rules (right).

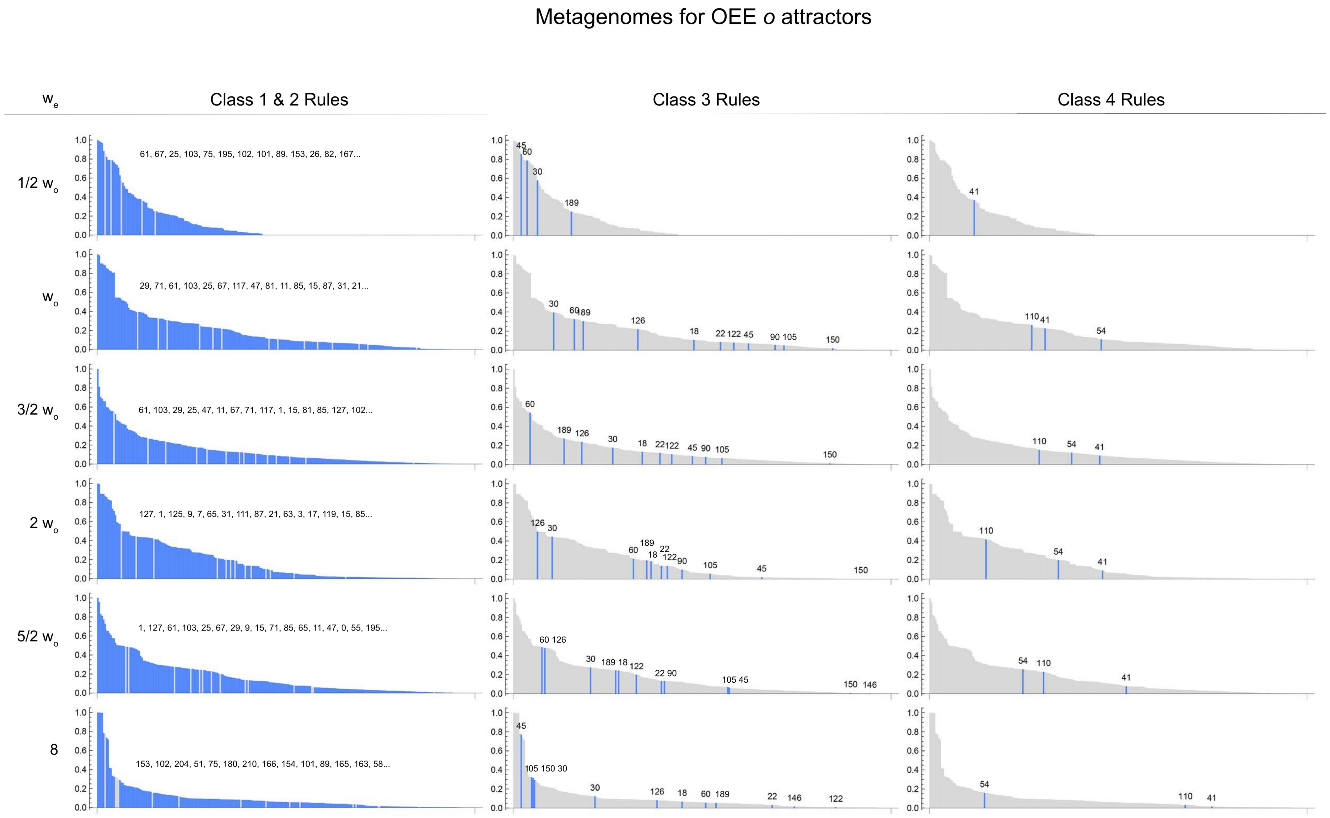

Figure 6. Rank ordered frequency distributions of rules ("metagenomes") implemented by o in its attractor for OEE cases only. From top to bottom $w_e = \frac{1}{2}w_o$, w_o, $\frac{3}{2}w_o$, $2w_o$ and $\frac{5}{2}w_o$, respectively. Highlighted in blue are the frequencies of Class I and II rules (left), Class III rules (middle) and Class IV rules (right).

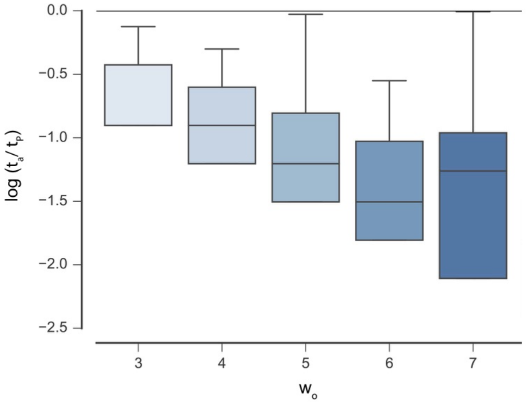

Figure 7. Distribution of attractor sizes t_a for the state trajectory of for all 88 non-equivalent ECA rules, evolved from all possible initial conditions of width w_o. Attractor sizes are normalized to the Poincaré time $t_P = 2^{w_o}$ for an isolated ECA, where the black horizontal line indicates where $t_r/t_P = 1$ (shown on a log scale).

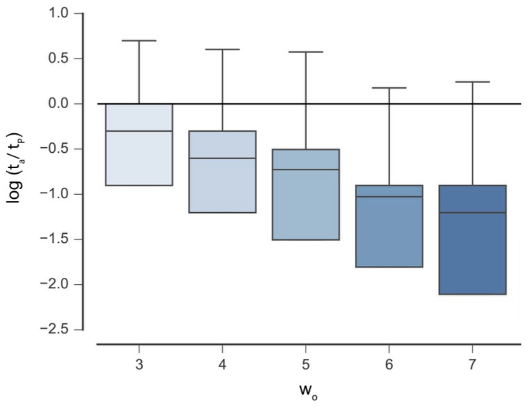

Figure 8. Distribution of attractor sizes t_a for the state trajectory of o, for Case II CA. Attractor sizes are normalized to the Poincaré time $t_P = 2^{w_o}$ for an isolated ECA. The black horizontal line indicates where $t_a/t_P = 1$ (shown on a log scale). Sample trajectories displaying *unbounded evolution* (UE) occur for $t_a/t_P > 1$.

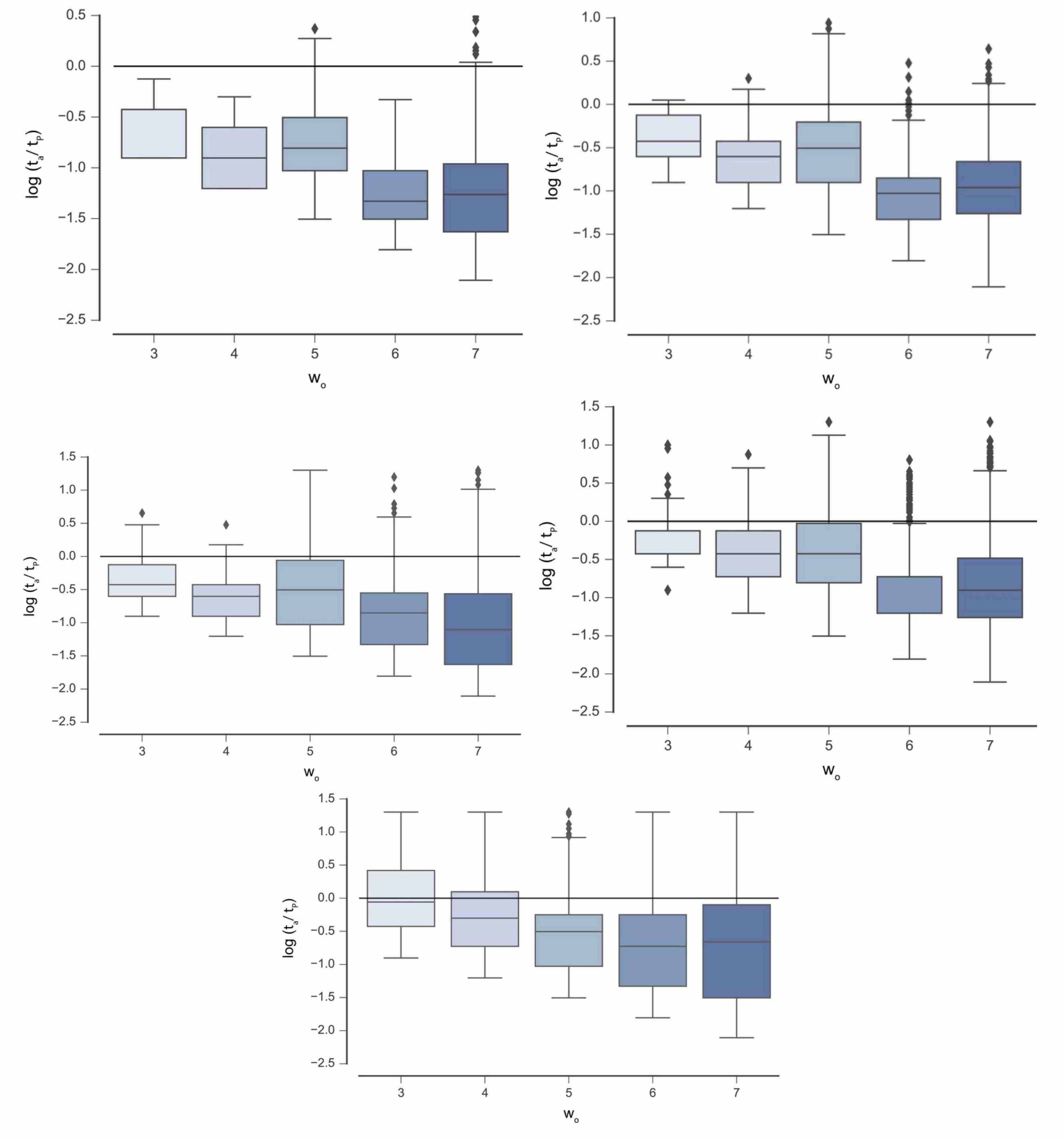

Figure 9. Distribution of attractor sizes t_a for the state trajectory of o for Case I CA. Shown from top to bottom are distributions for $w_e = \frac{1}{2}w_o$, w_o, $\frac{3}{2}w_o$, $2w_o$ and $\frac{5}{2}w_o$, respectively. Attractor sizes are normalized to the Poincaré time $t_P = 2^{w_o}$ for an isolated ECA. The black horizontal line indicates where $t_a/t_P = 1$ (shown on a log scale). Sampled trajectories displaying UE occur for $t_r/t_P > 1$.

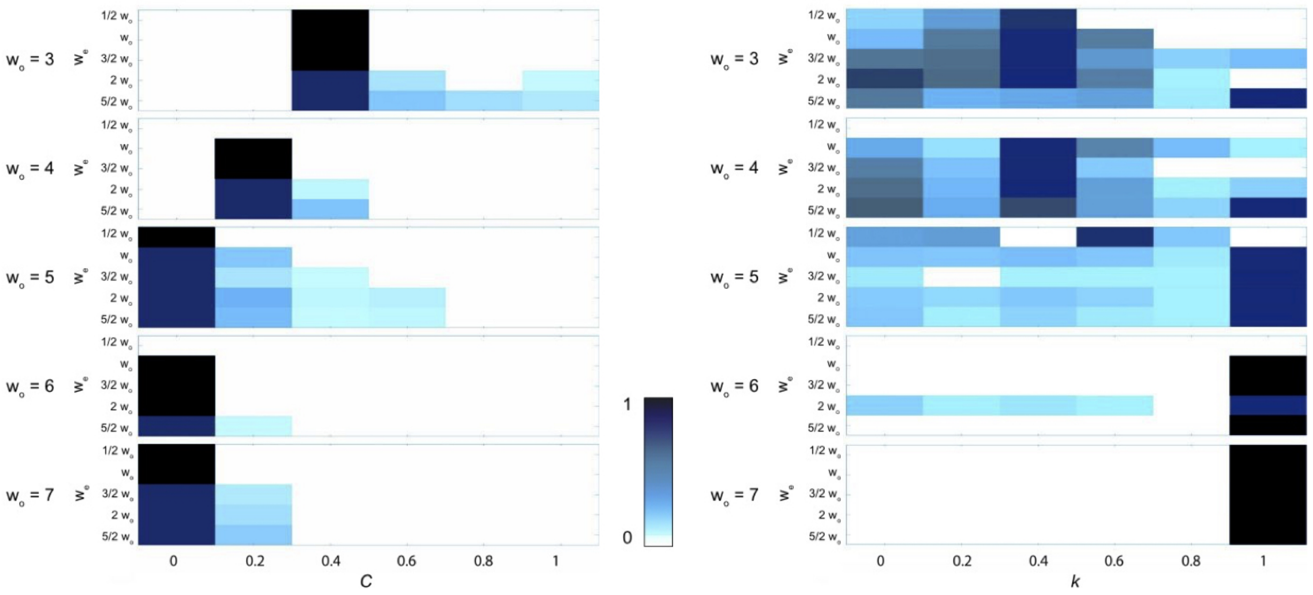

Figure 10. Heat maps of compression C (left) and Lyapunov exponent values k (right) for sampled OEE trajectories for the states of o for Case I CA. From top to bottom $w_o = 3,4,5,6$ and 7, with distributions shown for $w_e = \frac{1}{2}w_o$, w_o, $\frac{3}{2}w_o$, $2w_o$ and $\frac{5}{2}w_o$ (from top to bottom, respectively) for each w_o. Distributions are normalized to the total size of sampled trajectories for each w_o and w_e (see statistics in Table 1).

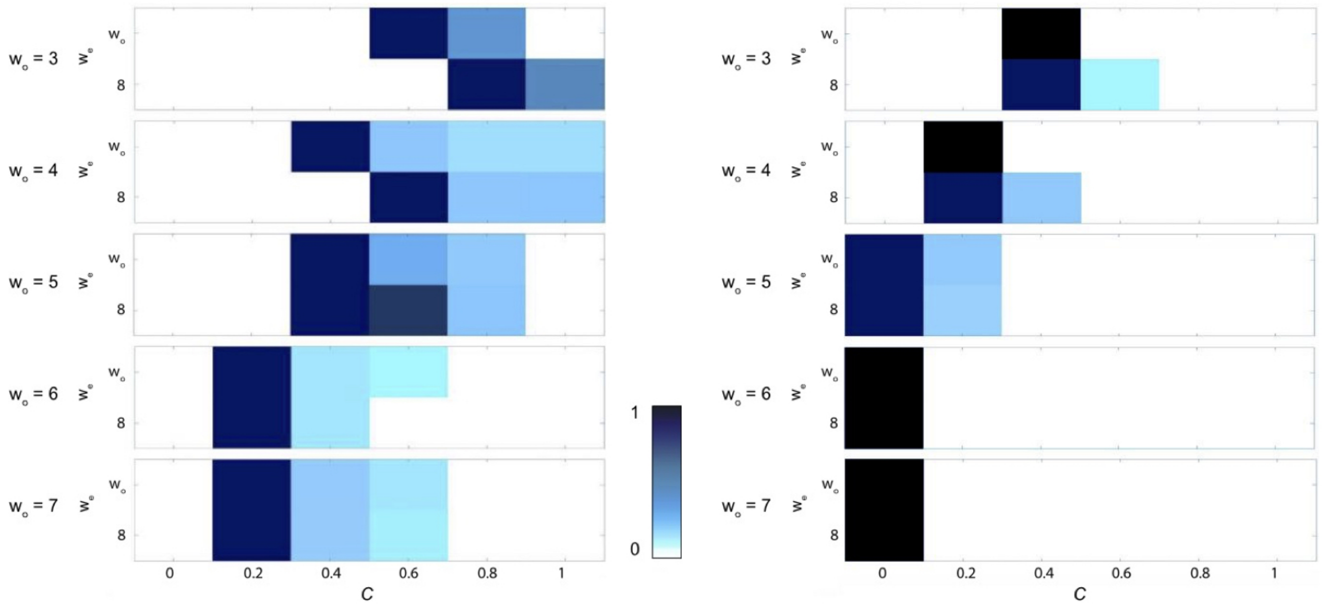

Figure 11. Heat maps of compression C for all sampled trajectories of the states of o (left), and for OEE trajectories only (right) shown for Case I and Case II CA. From top to bottom $w_o = 3,4,5,6$ and 7. For each w_o shown are the distributions of C for Case I CA for $w_e = w_o$ (top row in each panel) and for Case II CA with $w_e = 8$ (bottom row in each panel). Distributions are normalized to the total size of sampled trajectories for each w_o and w_e for each CA variant (see statistics in Tables 1 and 2).

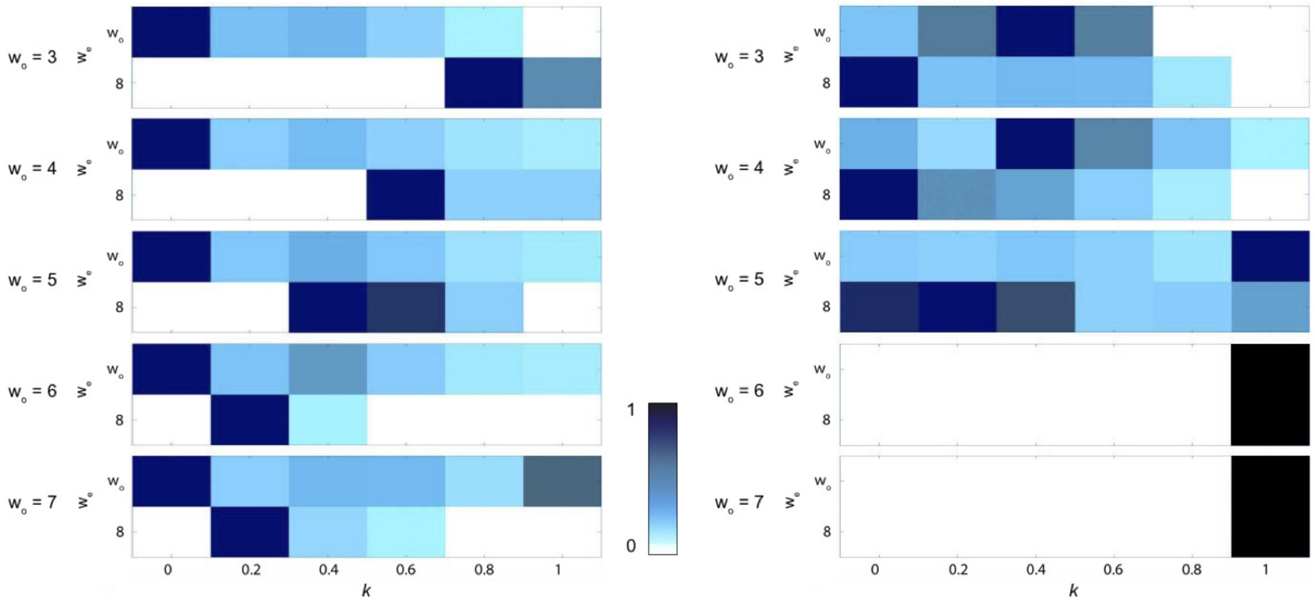

Figure 12. Heat maps of Lyapunov exponent k for all sampled trajectories of the states of o (left), and for OEE trajectories only (right) shown for Case I and Case II CA. From top to bottom $w_o = 3, 4, 5, 6$ and 7. For each w_o shown are the distributions of k for Case I CA for $w_e = w_o$ (top row in each panel) and for Case II CA with $w_e = 8$ (bottom row in each panel). Distributions are normalized to the total size of sampled trajectories for each w_o and w_e for each CA variant (see statistics in Tables 1 and 2).

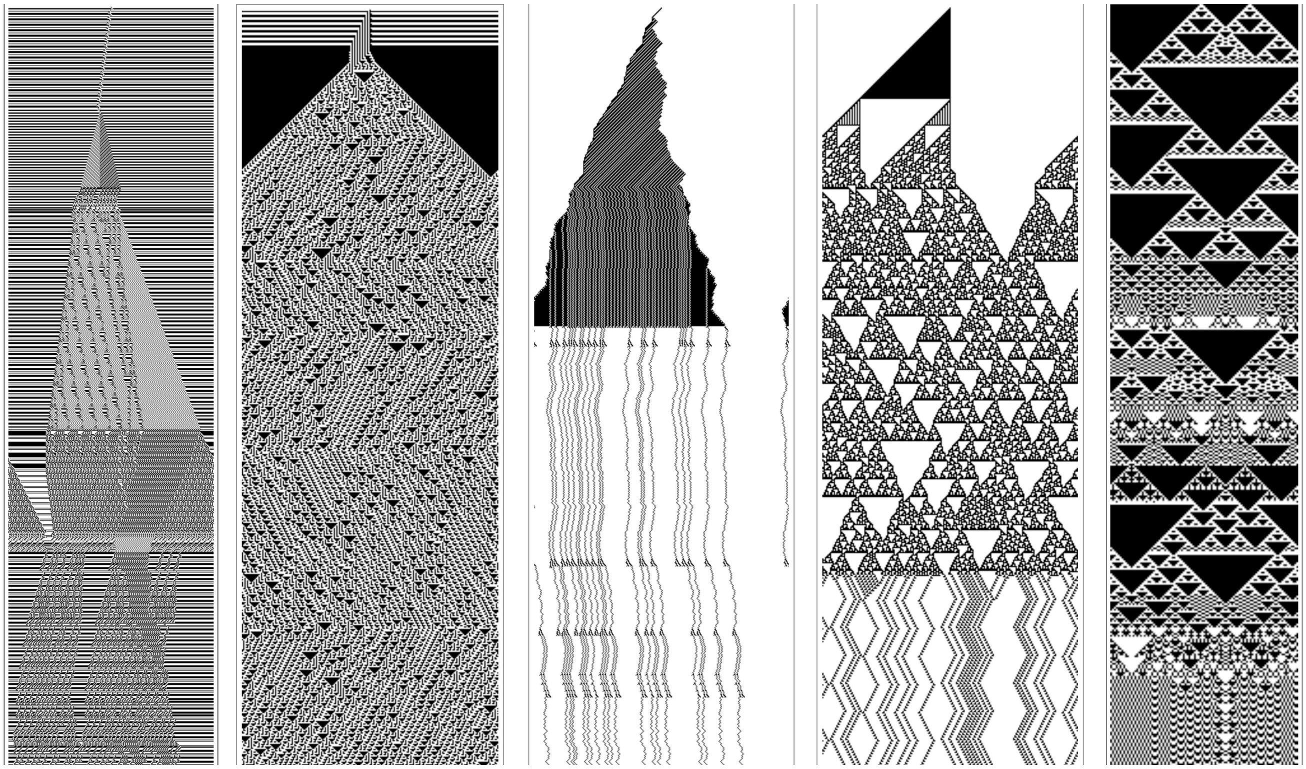

Figure 13. Example executions of the state trajectory of o for Case I CA for large system size $w_o = 101$, with $w_e = w_o$.
